# Tumeur phyllode maligne du sein après maladie de Hodgkin

**Published:** 2012-12-20

**Authors:** El Hfid Mohamed, Mezouar Loubna, El Harroudi Tijani

**Affiliations:** 1 Faculté de Médecine et de Pharmacie, Université Mohammed Premier, Oujda, Maroc; 2 Centre régional d’'oncologie Hassan 2, Oujda, Maroc

**Keywords:** Tumeur phyllode, maladie de Hodgkin, radiochimiothérapie, phyllodes tumor, Hodgkin disease, radiochimiotherapy

## Abstract

Le traitement par radiochimiothérapie de la maladie d'Hodgkin a permis une amélioration spectaculaire de son pronostic. Cependant, les néoplasies secondaires à ce type de traitement sont fréquentes et représentent un sérieux problème. Nous rapportons un cas de tumeur phyllode maligne du sein, diagnostiquée 15 ans après le traitement d'une maladie d'Hodgkin, en discutant ses différentes particularités diagnostiques et thérapeutiques, ainsi que les stratégies de suivi et de surveillance optimales.

## Introduction

La maladie d'Hodgkin (MH) a été décrite la première fois en 1832. Il fallait attendre ces derniers 30 ans pour assister à une réelle avancée aussi bien dans le diagnostic que le traitement de cette maladie; avec un taux de guérison actuellement qui avoisine les 85% [1]. Cependant, le suivi à long terme des ces patients traités par radiothérapie et chimiothérapie avait révélé une augmentation très significative des cancers secondaires [1–3]. Nous rapportons ici un cas de tumeur phyllode maligne du sein diagnostiquée 15 ans après le traitement par chimiothérapie et radiothérapie d'une MH.

## Patient et observation

Une femme de 39 ans a été vue en notre consultation le 28 Juin 2011; Il s'agit d'une célibataire, de niveau socioéconomique bas, sans profession. Elle a présenté il y a 15 ans une maladie d'Hodgkin sus et sous diaphragmatique classée stade 4 (atteinte du péricarde et de la plèvre gauche). Le traitement selon les standards de l’époque (1996) a consisté en six cures de chimiothérapie première type COPP-ABV alterné (cyclophosphamide, oncovin, procarbazine, et prednisone/Adriamycin, bleomycin et vinblastine), puis une radiothérapie de clôture par Télécobalt de 36 Gy en sus et sous diaphragmatique (“Mantelet” et “Lomboaortique + rate”) et un complément jusqu’à 46,8 Gy sur l'atteinte péricardique et pleurale. Les consultations de suivi ultérieures n'ont pas décelé de signes de récidive et la patiente a été considérée comme guérie.

A la consultation d'admission, la patiente rapporte une mastodynie, associée à l'autopalpation d'un nodule du sein droit retro-mamelonaire augmentant rapidement de volume.

**Figure 1:**
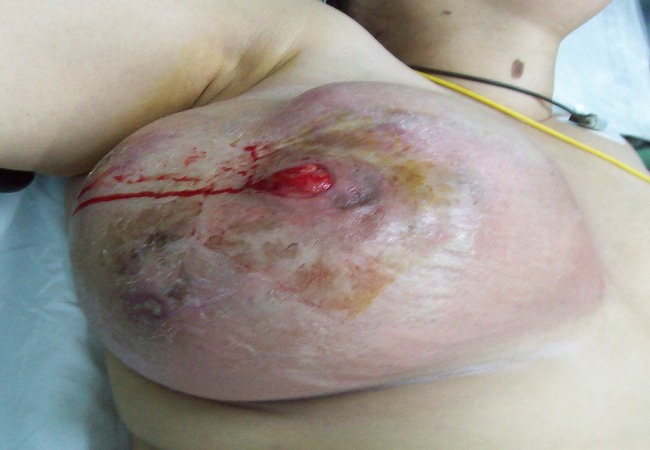
Tumeur bosselée prenant la totalité du sein droit avec ulcérations cutanées

L'examen clinique retrouve une masse bosselée, ulcérée à la peau et prenant tout le sein droit, associée à des adénopathies axillaires homolatérales mobiles (Figure 1).

La mammographie parle d'une opacité de tout le sein droit, hyperdense, bien limitée, sans microcalcifications. Le complément échographique retrouve une formation à contours arrondis, lobulée, hypoéchogène, hétérogène, associée à de multiples adénopathies axillaires homolatérales.

La cytoponction de la tumeur mammaire est en faveur d'une tumeur phyllode.

**Figure 2:**
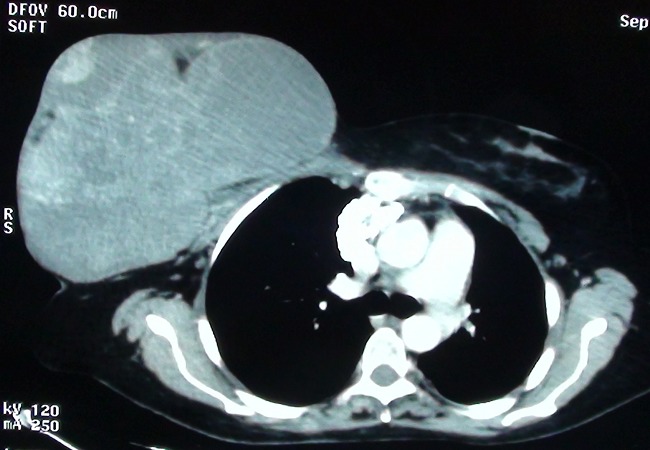
Coupe axiale d'un scanner thoracique montrant un volumineux processus tumoral mammaire droit, avec une double composante liquidienne et tissulaire

Le scanner thoraco-abdomino-pelvien réalisé dans le cadre du bilan d'extension tumoral ne retrouve pas de métastases à distance, et met en évidence le volumineux processus tumoral mammaire droit mesurant 22 centimètres de grand axe, avec une double composante liquidienne et tissulaire mais sans signes d'extension aux structures de voisinage (Figure 2).

La patiente a bénéficié par la suite d'une mastectomie radicale droite élargie avec curage axillaire de nécessité. Le compte rendu anatomo-pathologique conclut à une exérèse in sano d'une tumeur phyllode maligne de 22 centimètres, les ganglions du curage ainsi que la peau et le mamelon étaient tous indemnes d'envahissement tumoral.

Les suites opératoires étaient simples, et la patiente est toujours en bon contrôle locorégional huit mois après le geste chirurgical.

## Discussion

Le traitement par l'association chimiothérapie et radiothérapie des patients atteints de la MH avait, certes, permis une amélioration spectaculaire de son pronostic et de guérir presque 85% des patients. Cependant, on a constaté l'apparition à long terme de néoplasies secondaires, les cancers du sein en représentent 6 à 9% [2]. En fonction de l’âge de la patiente et des paramètres du traitement, le sur-risque de présenter un cancer du sein secondaire peut atteindre jusqu’à 136 fois celui de la population générale [1].

L’âge jeune au moment du traitement de la MH représente un facteur de risque essentiel; en effet, les patientes de moins de 20 ans présentent un risque relatif (RR) de 56 de développer un cancer du sein secondaire [2]. Notre patiente a été diagnostiquée et traitée à l’âge de 24 ans et selon plusieurs études, tout âge inférieur à 30 ans confère un risque accru de cancer du sein secondaire [2]. Le traitement par une radiothérapie thoracique à une dose supérieure à 40 Gray, comme a été le cas pour notre patiente, représente lui aussi un facteur de risque majeur [3].

Les traitements reçus par notre patiente correspondaient à ceux en vigueur au moment de sa MH, mais ne sont plus conformes aux référentiels actuels. Les traitements de la MH évoluent vers une désescalade thérapeutique (réduction des indications et des doses de radiothérapie et de chimiothérapie) qui pourrait modifier le risque de néoplasie secondaire [2].

Le délai d'apparition des néoplasies mammaires après le traitement initial de la MH est d'une importance capitale pour décider des modalités du suivi adéquates. Ce temps de latence moyen est aux alentours de 15 ans [1] et coïncide parfaitement avec celui de notre patiente. Les modalités de surveillance d'une patiente traitée pour une MH font débat. À la vue des données épidémiologiques, cette surveillance devrait débuter dès la dixième année après la fin des traitements [4]. Le National Comprehensive Cancer Network [4] propose un examen clinique annuel avant 25 ans, puis bi-annuel après 25 ans selon les données cliniques et le risque individuel. Le dépistage doit être débuté dès dix ans après la ‘n de l'irradiation et/ou à partir de 40 ans, et comporter une mammographie annuelle éventuellement associée à une imagerie par résonance magnétique (IRM). Le gold standard en imagerie varie selon les équipes. Dans notre contexte de pays en voie de développement, la mammographie doit rester l'examen de première intention. Des études récentes [5, 6], montrent que l'IRM, quand elle est disponible, pourrait être intéressante chez ces patientes à haut risque, d'autant qu'elle n'est pas irradiante et est performante chez des patientes jeunes aux seins denses.

La particularité de notre cas réside essentiellement dans le type histologique “tumeur phyllode maligne”, car en dehors d'une seule publication [7], tous les cas rapportés dans la littérature sont des carcinomes. Le traitement des tumeurs phyllodes mammaires est chirurgical, les traitements adjuvants ont peu d'intérêt [8].

La mastectomie simple était pendant longtemps le traitement standard des tumeurs phyllodes malignes. Actuellement, la plupart des auteurs réserve ce geste mutilant aux tumeurs trop grosses prenant tout le sein; ceci dans le but d'obtenir une marge d'exérèse saine d'au moins un centimètre. Le curage axillaire ne peut se concevoir qu'en cas d'adénopathies axillaires palpables suspectes cliniquement [8–10].

Dans notre cas, la réalisation d'une mastectomie radicale a permis d'avoir une exérèse in sano malgré la taille importante de la tumeur mammaire (22 cm). Devant la palpation d'adénopathies axillaires suspectes, un curage axillaire de nécessité a été réalisé mais n'a pas mis en évidence d'envahissement ganglionnaire.

A prendre en considération le caractère malin de la tumeur et la taille tumorale importante, l'indication d'une radiothérapie adjuvante peut être retenue. Cependant, l'absence d'impact sur la survie de cette modalité thérapeutique [9] et l'antécédent d'irradiation antérieur sur le même site à une dose supérieure à 40 Gray, nous ont incités à opter plutôt pour une surveillance active. D'autant plus que la chimiothérapie et l'hormonothérapie adjuvante n'ont pas démontré un intérêt particulier dans ce type d'indication [8].

## Conclusion

La prévention des cancers secondaires au traitement de la MH passe certainement par le processus déjà entamé de la désescalade thérapeutique. Mais, en attendant de pouvoir constater l'impact de ce processus sur la survenue de néoplasies secondaires, toute femme jeune traitée pour MH doit bénéficier d'une surveillance clinique, mammographique voire une IRM mammaire, de façon précoce dont la fréquence reste à définir et cela au minimum dès la dixième année après la fin du traitement. Ceci afin de détecter et traiter ces tumeurs à un stade précoce.
